# The Effects of the World Trade Center Event on Birth Outcomes among Term Deliveries at Three Lower Manhattan Hospitals

**DOI:** 10.1289/ehp.7348

**Published:** 2004-09-08

**Authors:** Sally Ann Lederman, Virginia Rauh, Lisa Weiss, Janet L. Stein, Lori A. Hoepner, Mark Becker, Frederica P. Perera

**Affiliations:** ^1^Columbia Center for Children’s Environmental Health, Mailman School of Public Health, Columbia University, New York, New York, USA; ^2^Department of Obstetrics and Gynecology, Beth Israel Medical Center, New York, New York, USA; ^3^Center for International Earth Science Information Network, Columbia University, New York, New York, USA

**Keywords:** birth length, birth weight, geographic information systems, gestational duration, head circumference, newborns, World Trade Center

## Abstract

The effects of prenatal exposure to pollutants from the World Trade Center (WTC) disaster on fetal growth and subsequent health and development of exposed children remain a source of concern. We assessed the impact of gestational timing of the disaster and distance from the WTC in the 4 weeks after 11 September on the birth outcomes of 300 nonsmoking women who were pregnant at the time of the event. They were recruited at delivery between December 2001 and June 2002 from three hospitals close to the WTC site. Residential and work addresses of all participants for each of the 4 weeks after 11 September 2001 were geocoded for classification by place and timing of exposure. Average daily hours spent at each location were based on the women’s reports for each week. Biomedical pregnancy and delivery data extracted from the medical records of each mother and newborn included medical complications, type of delivery, length of gestation, birth weight, birth length, and head circumference. Term infants born to women who were pregnant on 11 September 2001 and who were living within a 2-mile radius of the WTC during the month after the event showed significant decrements in term birth weight (−149 g) and birth length (−0.82 cm), compared with infants born to the other pregnant women studied, after controlling for sociodemographic and biomedical risk factors. The decrements remained significant with adjustment for gestational duration (−122 g and −0.74 cm, respectively). Women in the first trimester of pregnancy at the time of the WTC event delivered infants with significantly shorter gestation (−3.6 days) and a smaller head circumference (−0.48 cm), compared with women at later stages of pregnancy, regardless of the distance of their residence or work sites from the WTC. The observed adverse effects suggest an impact of pollutants and/or stress related to the WTC disaster and have implications for the health and development of exposed children.

At the time of the World Trade Center (WTC) tragedy, scientists and community members raised concerns about the effects on pregnant women and their children of exposure to the dust, smoke, and fumes. Analysis of dust samples from lower Manhattan in the days after the WTC event yielded a wide range of toxicants and irritants from building debris and combustion products (Lioy et al. 2002; McGee et al. 2003; Offenburg et al. 2003). These included a variety of neurodevelopmental toxicants and carcinogens such as polycyclic aromatic hydrocarbons (PAHs), polychlorinated biphenyls (PCBs), polychlorinated dibenzodioxins, polychlorinated dibenzofurans, pesticides, other hydrocarbons, and metals (Chen and Thurston 2002; Jeffrey et al. 2003; Lioy et al. 2002; McKinney et al. 2002; Offenburg et al. 2003). The WTC plume contained high levels of PAHs, levels that spiked 1.8 km (1.1 mile) northeast of the WTC site several times in September and October 2001, with a peak on 3 October, during an inversion that brought smoke back to ground level. Similarly, measurements of trace elements, including lead, taken five blocks from the WTC site spiked in September and October (Service 2003), indicating variability in ambient exposures throughout the month after the event.

The fetus is thought to be more sensitive than the adult to a range of ambient pollutants, including PAHs, with recent studies showing a higher rate of genetic damage from PAHs and slower clearance of other toxicants in the newborn compared with the mother (Perera et al. 2003). Fetal growth effects of PAHs and other pollutants have been demonstrated (Dejmek et al. 1999, 2000; Perera et al. 1998; Wilhelm and Ritz 2003); specifically, prenatal exposure to airborne PAHs was associated with reduced birth weight, birth length, and head circumference (Perera et al. 1998, 2003). Shortening of gestation has also been shown for cigarette smoke (Centers for Disease Control and Prevention 2002; Lewtas 1994), of which PAH is only one of many constituents. At the areal level, previous studies of PCBs and ambient carbon monoxide show that birth weight is associated with distance from the source of the pollutants or the site of their measurement (Baibergenova et al. 2003; Ritz and Yu 1999), suggesting that geographic location can be used to approximate level of exposure.

In addition to concerns about the potentially high levels of exposures from the WTC event, questions arise as to possible differential risk associated with the timing, during gestation, of pregnant women’s exposure to the event itself and to the air pollutants associated with the event. One study found a significant association between early gestational exposure to particulate matter (or an associated air pollutant) and intrauterine growth retardation (Dejmek et al 1999). Other studies have demonstrated that women exposed to active and/or passive smoking (environmental tobacco smoke exposure) throughout pregnancy show significantly larger birth weight decrements compared with women who reduced exposure early in the pregnancy (Dejmek et al. 2002).

The WTC disaster was a singular event and highly stressful for many individuals. Several previous studies have demonstrated effects of stressful conditions on birth outcomes such as preterm delivery and low birth weight (Arck et al. 2001; Axelrod and Reisine 1984; Da Costa et al. 2000; Dunkel-Schetter 1998; Glynn et al. 2001; Goepfert and Goldenberg 1996; Hobel et al. 1999; Wadhwa et al. 1998, 2001). There is also some emerging evidence suggesting that stressful exposures may exacerbate the impact of toxicants (Rauh et al. 2004; Singer et al. 2002).

We undertook the present study to assess the birth outcomes of women in the New York City area who lived or worked in the vicinity of the WTC at the time of the WTC event and during the weeks that followed. Future analyses will use biomarker data on individual air pollutants (including PAHs, PCBs, dioxins, furans, brominated fire-retardant breakdown products, lead, cadmium, and mercury) measured in maternal blood and urine and cord blood.

## Materials and Methods

### Recruitment sites.

Three large downtown hospitals with maternity units were selected based on their close proximity to the WTC site and the characteristics of their local catchment areas. These were Beth Israel and St. Vincent’s hospitals (and St. Vincent’s affiliated Elizabeth Seton Childbearing Center), all approximately 2 miles from the WTC site, and New York University (NYU) Downtown Hospital, which is within a half-mile of the site. These three hospitals deliver a total of nearly 9,000 infants each year. All three draw women from Manhattan, but each also serves a broader area that includes the outlying boroughs as well as parts of New Jersey and Westchester, New York. Because of the ready availability of bilingual staff (Chinese/English) at NYU Downtown, most of their deliveries are to Chinese women residing in Chinatown in lower Manhattan as well as the wider catchment area.

### Sample selection.

Singleton pregnant women were approached for enrollment by project staff at the time of labor and delivery at each of the three participating hospitals. Eligible women were between 18 and 39 years of age, met geographic criteria (described below), had not smoked during pregnancy (< 1 cigarette/day at any time), and reported no diabetes, hypertension, HIV infection or AIDS, or use of illegal drugs in the last year. Enrollment occurred between 13 December 2001 and 26 June 2002 and was implemented at each of the participating hospitals as soon after 11 September as institutional review board approval was obtained. Women were briefly screened for eligibility, recruited, enrolled, and confirmed as consenting while they were in labor, and interviewed after delivery by bilingual interviewers in their preferred or native language (English, Spanish, or Chinese).

Of 738 women initially screened, 369 women were eligible and gave consent for participation, 240 women were not eligible for participation, and 129 women refused to participate. Of the 369 eligible women, 329 contributed at least one blood sample (cord or maternal blood), medical record information, and a complete postpartum interview, all of which were required for full enrollment in the study. The sample was further reduced to 300 by the subsequent exclusion of women who were not yet pregnant on 11 September 2001 and those who delivered preterm (described below).

### Data collection.

Information about the pregnancy and delivery was collected from the medical records of the mother and newborn. A 30- to 45-min interview was administered to each mother, in most cases on the day after delivery. The interview included questions on demographics, reproductive history, background environmental exposures, where the woman was working or living (where she actually was, even if not her usual residence or place of work) during each of the 4 weeks after 11 September 2001 and the average hours per day spent at each site in each week. Biomedical information about the pregnancy, type of delivery, and birth outcomes (gestational duration, birth weight, birth length, head circumference) were abstracted from the medical record or obtained from the maternal interview if the medical record was incomplete. The week of gestation at which the WTC event occurred was determined for all women. Because the timing of the event might have had an effect on the type and extent of any fetal growth problems associated with the event, we included a variable indicating whether or not the participant was in the first trimester on 11 September 2001. Because hospital recruitment did not begin until 13 December 2001, because of the need to obtain institutional review board approvals from the participating hospitals, women who delivered between 11 September 2001 and 13 December 2001 were not included in the study. Most of these excluded women were in the later stages of pregnancy at the time of the disaster; hence, most of the participants were in their first or second trimester on 11 September.

### Geographic data.

Residential and work (for employed women) addresses were geocoded at the Center for International Earth Science Information Network of Columbia University’s Earth Institute, using geographic information system (GIS) software from Environmental Systems Research Institute (Redlands, CA), including ArcGIS 8.3 and the Street Map 2003 extension. The Manhattan hydrology layer came from the NYC DEP-funded NYCMap Project (NYC Department of Information Technology and Telecommunications 1999). Using the geocoded data, the linear distance from the WTC site was computed for each residence and work site. The estimated level of horizontal accuracy of the geocoded location is ± 25 feet. The GIS data enabled us to locate women in each of the 4 weeks after the disaster.

### Statistical analyses.

We generated descriptive statistics and bivariate associations for all variables and examined them for distributional normality. We used multiple linear regression and logistic regression to assess the effects of proximity to the WTC site and stage of pregnancy when the event occurred, on gestational duration, birth weight, birth length, head circumference, ponderal index, and sex-specific small size for gestational age (SGA) among term deliveries (Alexander et al. 1996). We excluded 10 women who were determined not to have been pregnant on 11 September 2001, based on the gestational age of the baby at birth and the date of delivery. In addition, only women who had completed 36 weeks and 6 days of gestation, or 258 days, and considered full term were included in the analyses, because preterm delivery may have other, complex etiologies. These criteria excluded a total of 29 women, for a final sample of 300 women.

We included race/ethnicity (Asian, African American vs. all others) and Medicaid status (marker for poverty) as covariates in all analyses because of their potentially confounding effects on the relationship between exposure and birth outcomes. Other relevant covariates included infant sex, maternal height, prepregnancy weight, parity (0, ≥1), maternal age in years, cesarean section, and maternal medical complications (including preeclampsia, placental abruption, hypertension, and diagnosis of diabetes during the pregnancy). Smoking was not included because enrollment criteria required that women be nonsmokers during their entire pregnancy. In this cohort, sexually transmitted disease and alcohol use (self-report by interview yielded very low use) were not significantly associated with exposure or birth outcomes (*p* > 0.05), and their inclusion did not alter effect sizes. Therefore, we excluded them to limit the number of independent variables. All analyses were done using SPSS version 11.5 (SPSS Inc., Chicago, IL).

## Results

### Description of the sample.

Table 1 shows the maternal and newborn characteristics of the sample (*n* = 300) included in the analysis. The study population was diverse, reflecting the mixed residential and commercial nature of lower Manhattan and the broader area served by the delivery hospitals—an area with a wide socioeconomic range of employment and housing conditions. Of the 300 participants, 44.7% were college graduates and 17.7% had not completed high school. More than 80% were married or cohabiting for more than 7 years, 58.0% were having their first child, and only two women had a parity > 3, both in the reference group. Forty-two percent self-identified as white, 33% Asian, 15.3% black/African American, and 9.7% “other” or unidentified race/ethnicity. Of the 283 participants who responded to the question on Hispanic identification, 61 (21.6%) identified as Hispanic, of which 23 also identified themselves as white, 14 black/African American, and 24 “other/unidentified” race.

### Classification of women by time and place.

**Figure 1:**
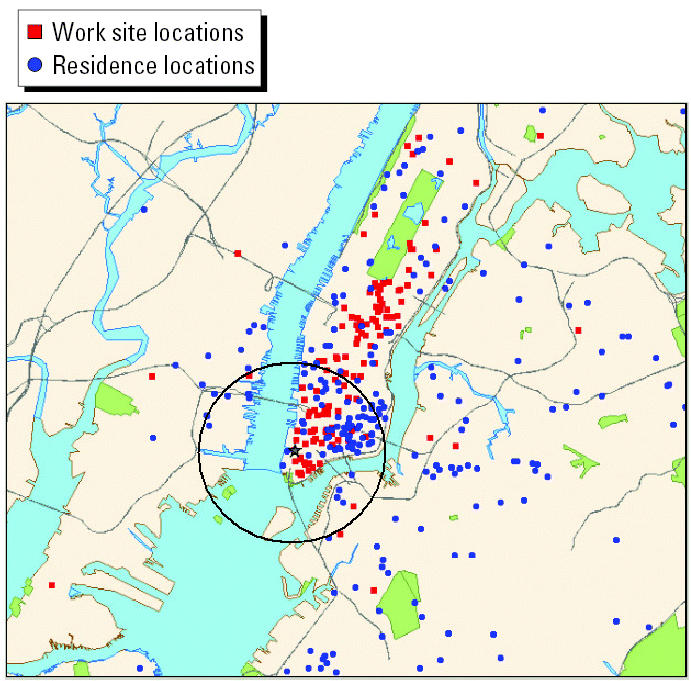
Geocoded locations of women’s work and residence addresses in the first week after 11 September 2001. Black circle indicates 2-mile radius from WTC site.

The wide distribution of geocoded locations of women’s residential and work sites during week 1 is illustrated in Figure 1. For the analyses, women who resided or worked within 2 miles of the WTC site, an area including sections of New Jersey and Brooklyn, New York, and indicated in Figure 1 by the circle, were selected to capture health effects of the smoke and emissions. Women were classified into three exposure groups. Women were first classified by residence location, grouping all women who were living within the 2-mile radius at some time in the 4 weeks after 11 September 2001, regardless of where they worked. Then the remaining women were classified by whether they worked but did not live within the 2-mile radius. Anyone not in either of these groups was in the reference group, women who neither worked nor lived within the 2-mile radius at any time during the 4-week period. Twenty women in the group that lived within 2 miles also worked within 2 miles of the WTC. They were classified as residents of the area. Depending on the week, between 17 and 21 women who resided within the 2-mile radius left home for some part of each day to travel to places of employment outside the 2-mile radius. These women were also classified with the resident group. In addition, we classified women as either being in the first trimester of pregnancy (≤91 days) on 11 September or being in a later trimester, based on their gestational age at delivery.

The sample of women who resided within the area remained very stable over the 4 weeks, with only three women moving in or out of the area during the time period. The number of women who worked but did not live within the 2-mile radius increased over the 4-week period as women gradually returned to work (24 in the first week, increasing to 51 women in the fourth week). In all but the first week, those who worked in the area were, on average, approximately 0.4 miles closer to the WTC site than were area residents.

**Figure 2:**
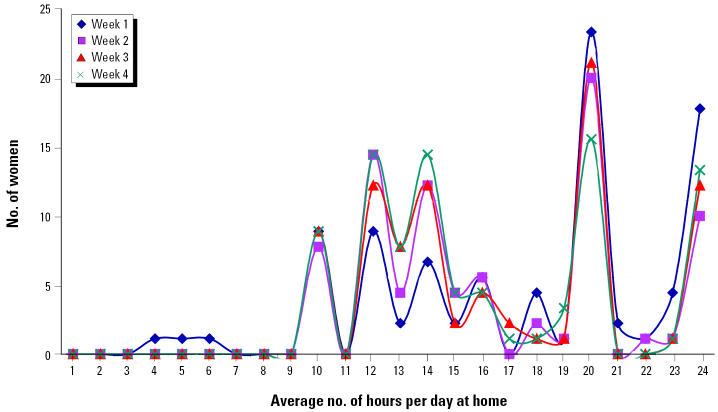
Average hours at home per day for women residing within 2 miles of the WTC site.

**Figure 3:**
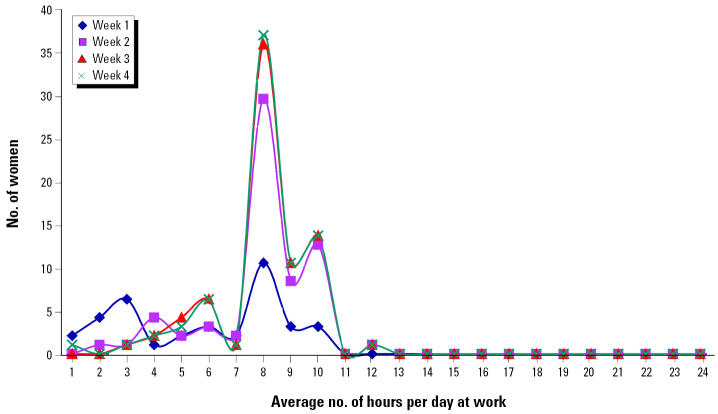
Average hours at work per day for women working within 2 miles of the WTC site.

The distributions of average daily hours at home or work site within the 2-mile radius for each week after 11 September are shown in Figures 2 and 3. Among women who resided in the area, daily hours of exposure were relatively stable over the 4 weeks, with a weekly average range of 16.2–17.1 hr/day. Among women who were employed in the area, the daily hours at work increased from an average of 5.9 hr in the first week to 8.0 hr in the fourth week, as normal work hours were gradually resumed. The overall distribution of hours was bimodal, resulting in a significant difference between the residential and employment groups in daily hours of exposure averaged over the 4-week period such that the residential group spent significantly more hours within the 2-mile radius (16.7 hr) compared with the employment group (7.7 hr).

### Birth outcomes.

All birth outcome analyses were limited to term deliveries. Table 2 shows the average length of gestation, birth weight, birth length, head circumference, ponderal index, and percentage of SGA births < 10th percentile; < 20th percentile is also shown) for term infants in each of the three exposure groups. There were significant group differences in unadjusted mean birth weight, birth length, and length of gestation. There were no significant differences in head circumference, ponderal index, or percentage of SGA births (either < 10th or < 20th percentile).

We used multiple regression analyses to assess the predictive power of proximity to the WTC and timing of the event in pregnancy for all birth outcomes, after adjustment for potential confounders and relevant covariates. Table 3, model 1, shows that term infants of women residing within the 2-mile radius during the 4 weeks after 11 September weighed, on average, 149 g less than did term infants born to women residing outside of that area, after adjustment for infant sex, maternal age, parity, prepregnancy weight, height, Medicaid receipt, race, and medical complications. This decrease in birth weight effect was not seen among infants born to women who were employed within the 2-mile radius.

The adverse effect on birth weight of residence within the 2-mile area was reduced but did remain significant after controlling for gestational duration (model 2), showing that the effect was partially mediated by but not entirely accounted for by shortening of gestation. The timing of the event during the first trimester of pregnancy was marginally significant (*p* = 0.057), and this effect was reduced substantially by the addition of gestational age to the model.

Table 3, model 3, shows the significant effect of residence within the 2-mile radius on birth length. Infants born to residents were, on average, 0.82 cm shorter than were those born to women living outside the area. This effect was reduced to 0.74 cm, yet remained significant, after the addition of gestational duration to the model (model 4). Again, this adverse effect was seen among area residents and not among women who traveled into the area to work in the first month. In fact, when gestation duration was controlled, women employed within 2 miles of the WTC had significantly longer infants (+ 1.0 cm). There was no significant birth length effect of trimester of pregnancy at the time of the event.

Table 4 shows that head circumference was not significantly associated with residence or employment within the 2-mile radius, after adjusting for covariates. There were significant negative effects on head circumference of timing of the WTC event, such that those who were in the first trimester of pregnancy on 11 September delivered term infants with significantly smaller heads (−0.48 cm). However, this effect was no longer significant with adjustment for gestational duration, indicating that the effect was mediated by shortening of duration of pregnancy. There were no residential or employment location effects on ponderal index or percentage of SGA births (< 10th percentile; results not shown).

Table 5 shows no significant reduction of length of gestation among term infants of women who lived within 2 miles of the WTC, and a marginally significant effect on those who worked in the area (*p* = 0.055). However, women who were in the first trimester of pregnancy on 11 September delivered, on average, 3.6 days earlier than did women in later stages of pregnancy, regardless of whether the women lived or worked close to the WTC. There were no significant interaction effects between distance and timing of exposure on any of the birth outcomes.

## Discussion

Our results indicate that term infants born to women who were living within 2 miles of the WTC site during any of the 4 weeks after 11 September 2001 had significantly lower birth weights and shorter birth lengths than did term infants born to women living outside of the area. These birth weight and length effects were only partially mediated by a shortening of gestation, suggesting some additional effect on fetal growth, independent of length of gestation. In addition, occurrence of the WTC event during the first trimester of pregnancy was associated with significantly shortened gestation and slightly smaller head circumference, regardless of place of work or residence in the month after 11 September. Head circumference effects were entirely explained by gestational duration.

Our findings of decreased birth weight and length among term infants of mothers who resided within 2 miles of the WTC site are potentially important for subsequent health and development. Lower birth weight, even within the normal range (> 2,500 g), is associated with increased fetal mortality (Seeds and Peng 2000), neonatal mortality (Arias et al. 2003; Rees at al. 1996; Seeds and Peng 2000), infant mortality (Arias et al. 2003), subsequent poorer health and delayed physical and cognitive development (Barker 1996; Dietz 1994; Matte et al. 2001; Rice and Barone 2000; Richards et al. 2002), and increased susceptibility to stress in adulthood (which decreases with increasing birth weight up to 4,200 g; Nilsson et al. 2001).

The lack of similar effects on birth weight or birth length of infants born to women who worked but did not reside within the 2-mile radius may be due to fewer hours of exposure in this group. As shown in Figures 2 and 3, women who worked in the 2-mile radius spent significantly fewer hours per day in the area than did residents. Furthermore, a number of the employed women did not return to work within the 2-mile radius during the first week and/or worked shorter hours in the first few weeks after 11 September 2001, possibly minimizing their exposure during the highest emission period. Those who traveled into the area to work for some hours each day returned home daily to residences located outside of the area.

The additional finding that exposure to the event during the first trimester of pregnancy was associated with significantly shorter length of gestation among term infants, regardless of location of work or residence, is clinically important. The last weeks and days of gestation are characterized by rapid fetal growth. First, it has been previously shown that birth weight increases by approximately 500 g between 37 and 41 weeks of gestation, and the increase in weight during this period of gestation is of similar magnitude for infants in all size for gestational age categories, such as the 10th, 50th, or 90th percentiles of size for gestational age (Alexander et al. 1996). As noted above, increased infant birth weight is associated with a range of improved newborn health and developmental outcomes (Matte et al. 2001). Second, a shortening of gestation from 41 to 37 weeks is associated with a 4-fold increase in fetal mortality (0.77 vs. 3.0 per 1,000 births) and a 4-fold increase in neonatal mortality (0.44 vs. 1.6 per 1,000 births) (Seeds JW, Peng TCC, unpublished data). Smaller changes in gestational duration would be expected to have proportionately smaller, but still potentially important, effects on these outcomes.

Several previous studies have shown significant adverse effects of exposure to air pollution during pregnancy on birth weight and other pregnancy outcomes. Among African Americans in New York City, prenatal exposure to PAHs, as measured by personal air sampling during pregnancy, was associated with reduced birth weight and head circumference (Perera et al. 2003). Similarly, a study of mothers and newborns in Poland found that cord blood PAH–DNA adducts were significantly inversely related to birth weight, length, and head circumference (Perera et al. 1998). Other air pollutants, including sulfur dioxide and particulate matter ≤10 μm in diameter have also been shown to be associated with birth weight decrements (Chen et al. 2002; Yang et al. 2003).

Other adverse birth outcomes associated with air pollution include preterm delivery (Ritz et al. 2000; Xu et al. 1995), low birth weight (Bobak 2000; Bobak and Leon 1999; Lin et al. 2001; Wang et al. 1997), intrauterine growth restriction (Dejmek et al. 1999), and birth defects (Ritz et al. 2002). In addition, studies have shown that prenatal exposure to air pollutants is associated with increased rates of neonatal and postneonatal mortality (Bobak and Leon 1999; Pereira et al. 1998; Woodruff et al. 1997) and infant mortality (Loomis et al. 1999).

The air pollution from the dust and gases emitted during the WTC event and subsequent cleanup operation resulted in a relatively brief exposure, yet it is likely that the exposure was substantial for those pregnant women who spent large portions of each day within 2 miles of the site during the month after the tragedy. Proximity to specific sources of pollution (or the site of their measurement) has been used previously to characterize exposure and has demonstrated adverse effects on birth weight (Baibergenova et al. 2003; Ritz and Yu 1999). Using a measure of traffic density weighted for distance from the home, Wilhelm and Ritz (2003) reported an increased risk of preterm delivery and low birth weight among women who resided in the nearest quintile of distance from heavily trafficked roads. Consistent with these reports, the effect on birth weight in our study was seen among women living close to the WTC site in the 4 weeks after 11 September.

Although residential proximity was associated with reductions in birth weight and length, there were no apparent distance effects on head circumference, ponderal index, or percent SGA. Head circumference and length of gestation, however, were significantly associated with the timing of the event in the first trimester of pregnancy, although the head circumference effect was accounted for by length of gestation. We found no effects of proximity to the WTC site in the 4 weeks after the event on the odds of SGA births (< 10th percentile). Using a different recruitment procedure, Berkowitz et al. (2003) have reported increased risk of SGA births (8.2%) in a sample of private patients who were near the WTC on 11 September or in the subsequent 3 weeks compared with a sample of private patients who delivered in northern Manhattan during the same period (3.8%).

In the present study, the findings that length of pregnancy was associated with timing of the event in pregnancy, regardless of proximity, and that the reduced gestational duration had measurable effects on birth weight and length, suggest that additional mechanisms operating early in pregnancy, possibly stress related, may have been operative. There were no interaction effects between proximity to the site and timing of exposure, although there is always the possibility that the fetus is most vulnerable to toxic biochemical exposures in the first few months.

With respect to stress-related hypotheses, a number of previous studies have linked maternal stressful exposures to preterm delivery (Dunkel-Schetter 1998; Goepfert and Goldenberg 1996; Hobel et al. 1999; Wadhwa et al. 1998) and reductions in birth weight (e.g., Da Costa et al. 2000). Recently, maternal stress has also been implicated in miscarriage, through effects on immune mediators (Arck et al. 2001). Other reports have addressed the role of the maternal stress response as a possible modulator of toxicant effects (e.g., Singer et al. 2002). Taken together, this literature provides a biologic basis for possible adverse effects of stressful exposures on length of gestation (e.g., Wadhwa et al. 2001). Glynn et al. (2001) have shown that exposure to an earthquake was perceived as most stressful by women in early pregnancy and less stressful by women in later stages. These researchers observed that exposure to the earthquake in the first trimester of women within 50 miles of the epicenter was associated with a shortening of gestation to 38.06 weeks, compared with 38.69 weeks in those exposed in the second trimester, a difference of 4.4 days. In our study, gestation was also shorter, by 3.6 days, among women who were in the first trimester on 11 September 2001 compared with all other women. Lobel et al. (1992) demonstrated reductions in birth weight as well as gestational duration associated with perceived stress/distress, but no association with SGA (< 10th percentile). As in the present study, shortening of gestation accounted for only part of the decrement in birth weight that was associated with stress, suggesting that chronic stress can affect birth weight directly as well as indirectly through effects on gestation length.

A major strength of the present study is enrollment of exposed and unexposed women from a common clinical population. All women delivered at one of three lower Manhattan hospitals during the same time period. In addition, enrollment occurred before delivery, before the outcome of the pregnancy was known, so women’s decision to participate was not based on their knowledge of their own birth outcome. The potential sample selection bias introduced by volunteerism of women who experienced worrisome birth outcomes was thus avoided. Recruitment procedures ensured a wide range of race/ethnicity, income, education, and other characteristics, improving the generalizability of our findings. In addition, the subjects were geographically well dispersed during pregnancy with respect to the WTC site, providing a basis for comparing subjects with differential exposure.

A limitation of the study is that, because of the time required to obtain institutional review board approval from the participating hospitals, recruitment did not begin until December. Thus, the sample did not include women who were exposed during the last trimester of pregnancy, and we were able to compare only first versus second trimester effects. We were also unable to assess preterm deliveries, stillbirths, or spontaneous abortions possibly resulting from toxic exposures. Despite the inability to study these women who may have been at excess risk, we did detect a significant reduction in gestational duration among term deliveries.

The results reported here show that pregnant women living close to the WTC after 11 September 2001 were at increased risk of delivering infants with reduced weight and length, and that newborns of women exposed to this event in the first trimester had shorter gestations regardless of distance from the WTC site. Planned evaluations of children in this cohort at 1 and 2 years of age will address additional questions about the longer-term health and development implications of maternal prenatal exposure to pollution and psychologic distress resulting from the WTC disaster.

## Figures and Tables

**Table 1:** Subject characteristics.

	No. of subjects	Mean ± SD	Range
Maternal age (years)	298	30.2 ± 5.14	18.1–40.7
Years of school	300	14.0 ± 3.58	2–21
Prepregnancy weight (lb)	299	135.7 ± 30.20	90–318
Height (cm)	298	163.1 ± 7.16	144.8–185.4
Income/household member*^a^*	272	$23,535 ± 17,053	$1,000–85,000
No. of prior live births*^b^*	300	0.62 ± 0.877	0–5
Primiparous (%)		58.0	
Birth weight (g)	300	3,454 ± 453.8	2,040–5,255
Birth length (cm)	291	50.9 ± 2.89	32.0–57.0
Head circumference (cm)	291	34.3 ± 1.49	29.0–38.0
Gestational age (days)	300	278.0 ± 8.43	259–297
Trimester on 11 September 2001			
First (< 92 days)	205		
Second (92–182)	92		
Third (≥183 days)	3		
1-Min Apgar	300	8.7 ± 0.82	2–10
5-Min Apgar	298	9.0 ± 0.32	6–10

a Income based on midpoint of each of 10 household income categories, ranging from < $10,000 to > $90,000. The midpoint of the first category was set at $5,000, and that of the last category was set to $95,000. Some women did not report income.

b Only two women had a parity > 3.

**Table 2:** Unadjusted birth outcomes by place of residence and employment (within 2 miles of the WTC).

Birth outcomes	Group 1: resided	Group 2: worked	Group 3: neither resided nor worked	*p*-Value*^a^*
Length of gestation (days)	277.7 (*n* = 80)	275.5 (*n* = 51)	279.0 (*n* = 169)	0.026
Birth weight (g)	3339.6 (*n* = 80)	3442.7 (*n* = 51)	3511.8 (*n* = 169)	0.019
Birth length (cm)	50.06 (*n* = 78)	51.44 (*n* = 48)	51.15 (*n* = 165)	0.008
Head circumference (cm)	34.10 (*n* = 78)	34.18 (*n* = 49)	34.51 (*n* = 164)	0.097
Ponderal index*^b^*	2.75 (*n* = 78)	2.54 (*n* = 48)	2.65 (*n* = 165)	0.286
Percent SGA (< 10th percentile)	8.75 (*n* = 80)	5.88 (*n* = 51)	5.33 (*n* = 169)	0.581
Percent SGA (< 20th percentile)	23.8 (*n* = 80)	15.7 (*n* = 51)	18.3 (*n* = 169)	0.465

a By analysis of variance.

b Values are (g/cm^3^) ×100.

**Table 3:** Multiple regression of birth weight and birth length on proximity to the WTC and timing of the event in pregnancy in a sample of lower Manhattan term deliveries, 13 December 2001 through 26 June 2002.

	Birth weight (g; *n* = 295*^a^*)				Birth length (cm; *n* = 287*^a^*)			
	Model 1		Model 2		Model 3		Model 4	
Predictor	Coefficient	*p*-Value	Coefficient	*p*-Value	Coefficient	*p*-Value	Coefficient	*p*-Value
Resided within 2 miles	−149	0.012	−122	0.024	−0.819	0.026	−0.737	0.039
Employed within 2 miles	1.44	0.984	53.7	0.419	0.853	0.063	1.01	0.024
1st trimester on 11 September	−104	0.057	−27.0	0.595	−0.204	0.545	0.075	0.823
Maternal age (years)	2.14	0.713	−2.24	0.675	0.021	0.569	0.006	0.859
Male infant	237	0.000	206	0.000	1.51	0.000	1.42	0.000
Parity (0, ≥1)	107	0.053	152	0.003	0.376	0.273	0.508	0.131
Prepregnancy weight (lb)	1.69	0.081	1.45	0.100	0.000	0.933	−0.002	0.795
Maternal height (cm)	10.5	0.011	13.6	0.000	0.085	0.001	0.095	0.000
Medicaid	110	0.090	55.4	0.352	0.981	0.015	0.790	0.045
Asian	45.0	0.482	23.4	0.690	−1.04	0.010	−1.12	0.004
Black	−60.8	0.424	−46.6	0.502	−0.901	0.060	−0.829	0.075
Maternal medical complications*^b^*	−136	0.146	−77.4	0.365	−2.18	0.000	−1.98	0.001
Length of gestation (days)			21.6	0.000			0.075	0.000

a Numbers vary slightly because of occasional missing data.

b Complications included were hypertension, diabetes, and preeclampsia. No women had placental abruption.

**Table 4:** Multiple regression of head circumference (cm; *n* = 286*^a^*) on proximity to the WTC and timing of the event in pregnancy in a sample of lower Manhattan term deliveries, 13 December 2001 through 26 June 2002.

	Model 1		Model 2	
Predictor	Coefficient	*p*-Value	Coefficient	*p*-Value
Resided within 2 miles	−0.288	0.151	−0.231	0.231
Employed within 2 miles	−0.156	0.527	−0.037	0.876
1st trimester on 11 September	−0.477	0.010	−0.300	0.096
Maternal age (years)	0.008	0.674	−0.002	0.902
Male infant	0.658	0.000	0.590	0.000
Parity (0, ≥1)	0.382	0.040	0.481	0.008
Prepregnancy weight (lb)	0.004	0.219	0.003	0.270
Maternal height (cm)	0.016	0.250	0.024	0.079
Medicaid	0.035	0.870	−0.084	0.692
Asian	−0.004	0.985	−0.048	0.819
Black	−0.279	0.280	−0.237	0.340
Maternal medical complications*^b^*	−0.286	0.377	−0.163	0.602
Cesarean-section	0.627	0.003	0.600	0.003
Length of gestation (days)			0.049	0.000

a Numbers vary slightly because of occasional missing data.

b Complications included were hypertension, diabetes, and preeclampsia. No women had placental abruption.

**Table 5:** Multiple regression of gestational duration (days; *n* = 298*^a^*) on proximity to the WTC and timing of the event in pregnancy in a sample of lower Manhattan term deliveries, 13 December 2001 through 26 June 2002.

	Length of gestation	
Predictor	Coefficient	*p*-Value
Resided within 2 miles	−1.22	0.279
Employed within 2 miles	−2.59	0.055
1st trimester on 11 September	−3.55	0.001
Male infant	1.26	0.188
Parity (0, ≥1)	−1.92	0.065
Medicaid	2.71	0.026
Maternal age (years)	0.19	0.089
Asian	1.42	0.216
Black	−1.04	0.462
Maternal medical complications*^b^*	−2.55	0.153

a Numbers vary slightly because of occasional missing data.

b Complications included were hypertension, diabetes, and preeclampsia. No women had placental abruption.
